# Survival Patterns of Human Prion Diseases in Spain, 1998–2018: Clinical Phenotypes and Etiological Clues

**DOI:** 10.3389/fnins.2021.773727

**Published:** 2022-01-20

**Authors:** Laura Tejedor-Romero, Teresa López-Cuadrado, Javier Almazán-Isla, Miguel Calero, Fernando J. García López, Jesús de Pedro-Cuesta

**Affiliations:** ^1^Department of Neurodegeneration, Ageing and Mental Health, National Centre for Epidemiology, Carlos III Institute of Health, Madrid, Spain; ^2^Preventive Medicine Unit, La Princesa University Teaching Hospital, Madrid, Spain; ^3^Consortium for Biomedical Research in Neurodegenerative Diseases (CIBERNED), Madrid, Spain; ^4^Alzheimer’s Disease Research Unit, Fundación CIEN (Centro de Investigación de Enfermedades Neurológicas), Queen Sofia Foundation Alzheimer Centre, Madrid, Spain; ^5^Chronic Disease Programme, Carlos III Institute of Health, Madrid, Spain

**Keywords:** survival, human spongiform encephalopathies, prognostic factors, clinical phenotypes, sporadic Creutzfeld-Jakob disease

## Abstract

**Background:**

Human transmissible spongiform encephalopathies (TSEs) are a group of fatal neurodegenerative disorders of short duration. There are few studies on TSE survival. This study sought to analyze the survival and related factors of a TSE patient cohort, based on a nationwide surveillance system in Spain.

**Methods:**

Survival analyses were performed on 1,530 cases diagnosed across the period 1998–2018 in Spain. We calculated median survival times and plotted survival curves using the Kaplan–Meier method for all cases and for sporadic TSE (sTSE) and genetic TSE (gTSE). Crude and adjusted Cox proportional hazard models were used to identify variables associated with shorter survival.

**Findings:**

Median age at onset decreased from the sporadic forms to gTSE and, lastly, to acquired TSE. Overall median and interquartile range (IQR) survival time was 5.2 (IQR, 3.0–11.7) months and 4.9 (IQR, 2.8–10.8) months in sporadic cases and 9 (IQR, 4.9 to over 12) months in genetic cases, *p* < 0.001. Male sex, older age at onset, presence of 14-3-3 protein, typical MRI, and MM and VV polymorphisms at codon 129 were associated with shorter survival. gTSE showed higher survival in crude comparisons but not after adjustment.

**Interpretation:**

TSE survival in Spain replicates both the magnitude of that shown and the TSE entity-specific population patterns observed in Western countries but differs from features described in Asian populations, such as the Japanese. The reduction in differences in survival between gTSE and sTSE on adjusting for covariates and international patterns might support the view that gTSE and sTSE share causal and pathophysiological features.

## Introduction

Human transmissible spongiform encephalopathies (TSEs) are a group of fatal neurodegenerative disorders caused by the abnormal disease-causing isoform (PrP^Sc^) of a normal cellular protein, i.e., the cellular prion protein (PrP^C^) (Prusiner, 1998). Annual incidence worldwide is estimated at 1–1.5 cases per million (Tee et al., 2018).

Among human prion diseases, 85–90% are sporadic Creutzfeldt–Jakob disease (sCJD) cases, 10–15% are inherited or genetic TSE [gTSE, including genetic Creutzfeldt–Jakob disease (gCJD), Gerstmann–Sträussler–Scheinker syndrome (GSS), or Fatal Familial Insomnia (FFI)], and less than 1% are acquired forms, either variant Creutzfeldt–Jakob disease (vCJD) or accidentally transmitted Creutzfeldt–Jakob disease (atCJD). vCJD is the only known zoonotic form of human prion disease and occurs through consumption of bovine tissues affected by bovine spongiform encephalopathy (BSE) and BSE-tainted blood transfusions (Geschwind, 2015). atCJD may appear as a consequence of treatment with human-derived growth hormone or gonadotropins and several homografts (Brown et al., 1992).

Prion diseases are characterized by a long incubation period, typically affect the central nervous system, and have a progressive and lethal course (Manzano et al., 2018). While clinical manifestations may vary in different forms of the disease, the most common manifestations include rapidly progressive cognitive impairment and dementia, behavioral symptoms, impairment in higher cortical functions, myoclonus in more than 90% of patients throughout the disease course, and akinetic mutism during the final stages (Brown Henry and Lee John, 2021). There can be significant variation in age at onset (Ironside et al., 2017).

Polymorphism in the prion gene, *PRNP*, at codon 129 is associated with survival, which is longer in heterozygous (MV) cases (Pocchiari et al., 2004). Plasma and cerebrospinal fluid (CSF) tau levels may be linked to survival (Staffaroni et al., 2019).

Knowledge of factors relating to survival might prove helpful in predicting the course of the disease, guiding clinical management and decision-making, and assessing the effectiveness of possible future treatments. To our knowledge, there are few studies on TSE survival (Puopolo et al., 2003; Pocchiari et al., 2004; Sanchez-Valle et al., 2004; Heinemann et al., 2007; Nagoshi et al., 2011; Iwasaki et al., 2015; Sun et al., 2020; Yang et al., 2020).

Accordingly, this study set out to analyze the survival and related factors of a TSE patient cohort, based on a nationwide surveillance system in Spain for 1993–2018 (De Pedro-Cuesta et al., 2021).

## Materials and Methods

### Data-Sources

This study consisted of a retrospective examination of records at the Spanish National Register of Human Transmissible Spongiform Encephalopathies. TSE surveillance in Spain began prospectively in 1995 when the National Register was created (Spain’s Ministry of Health and Consumption, 2020; Official State Gazette, 2021) and has also been conducted retrospectively until 1993. The register is kept by the National Centre of Epidemiology at the Carlos III Institute of Health, with surveillance units across all regions of Spain notifying suspected TSE cases to the Centre.

**FIGURE 1:**
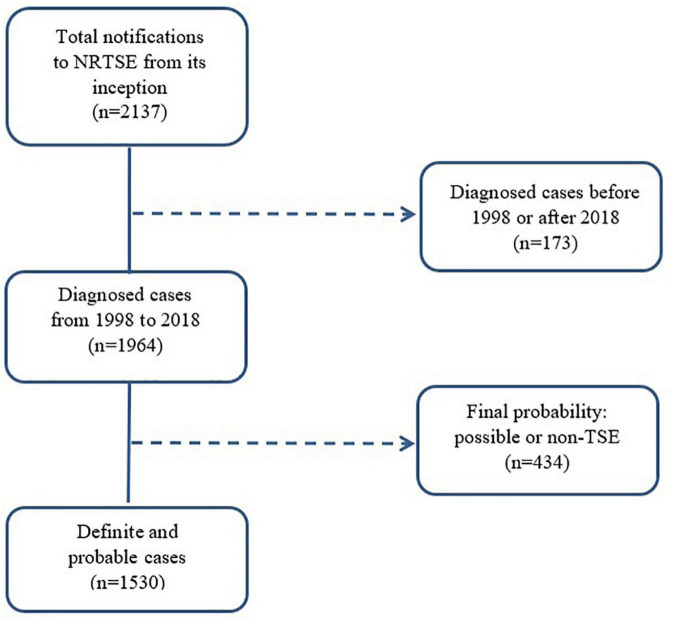
Flow chart showing case-selection for study purposes. NRTSE, National Register of Human Transmissible Spongiform Encephalopathies; TSE, Transmissible Spongiform Encephalopathy.

Among all cases notified to the register with diagnoses established during the period 1998–2018, we selected all those fulfilling criteria for probable and definite TSE, as laid down by the European Creutzfeldt–Jakob Disease Surveillance Network (EuroCJD). These criteria changed in 1998 when the use of the 14-3-3-protein test in CSF was introduced, in 2003 when epidemiological criteria (risk exposures) were included, and again in 2010 when magnetic resonance imaging (MRI) criteria were incorporated (Zerr et al., 2009). The most recent criteria update by the European Centre for Disease Prevention and Control was issued on January 1, 2017. The study accrual was 1,530 (see the flowchart in Figure 1 which describes the selection procedure).

### Variables

For analysis purposes, we used the following variables: sex; age in years at clinical onset (0–49, 50–59, 60–69, 70–79, and ≥ 80); presence of 14-3-3-protein in the CSF; polymorphism at codon 129 of the *PRNP* gene [methionine/methionine (MM), methionine/valine (MV), or valine/valine (VV)]; presence of periodic sharp wave complexes in electroencephalogram (EEG), high MRI signal in the caudate and putamen; form of clinical onset (rapidly progressive dementia or other); and type of TSE, i.e., sporadic or genetic (including gCJD, GSS, and FFI).

### Statistical Analysis

A descriptive analysis was performed for each form of TSE and all the variables of interest. Qualitative variables were expressed as absolute values and percentages, and continuous variables as median and interquartile range (IQR).

Survival analysis was performed by taking the reported date of clinical onset as the starting point. “Event” was defined as death during the first year; otherwise, the case was censored. We estimated median survival times and displayed survival curves using the Kaplan–Meier method for all cases, and for sTSE and gTSE. Curves were compared using the log-rank test.

Crude and adjusted Cox proportional hazard models were used to identify the variables associated with survival. Results were expressed as hazard ratios (HRs) with their corresponding 95% confidence intervals (95% CI). To evaluate the proportional hazards assumptions, the Schoenfeld residual test was performed. All analyses were performed using the STATA/SE 15 computer software package (StataCorp LLC, College Station, TX, United States).

### Ethics Approval

This is an observational study conducted in public health context that does not meet any of the criteria required for revision by a research ethics committee, as stated in the Biomedical Research Act (Ley 14/2007 de Investigación Biomédica). Data are obtained from an epidemiological surveillance registry where no informed consent is required for registration and notification is mandatory by law since 2001.

## Results

**FIGURE 2:**
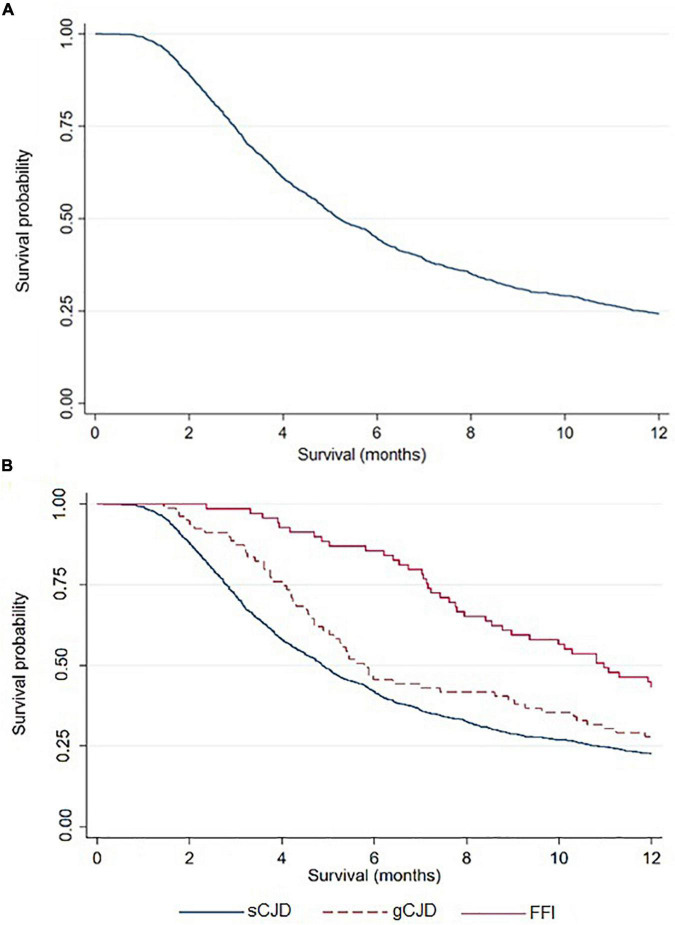
TSE Survival curve, overall **(A)** and by type **(B)**, using the Kaplan-Meier method.

The characteristics of 1,530 cases diagnosed between 1998 and 2018 are shown in Table 1: 46% were male, and median age at onset was 68 (IQR, 60–74) years. Acquired cases had the youngest age at disease onset, with a median of 47 (IQR, 40–64) years, followed by genetic cases with 53 (IQR, 46–62) years, whereas sporadic cases had a median age of 69 (IQR, 62–75) years at clinical onset. All vCJD and atCJD cases with available information were MM at codon 129. Overall median survival time was 5.2 (IQR, 3.0–11.7) months, with the breakdown showing 4.9 (IQR, 2.8–10.8) months for sporadic cases and 9 (IQR, 4.9 to over 12) months for genetic cases (Figure 2), *p* < 0.001.

**TABLE 1:** Characteristics of total definite and probable transmissible spongiform encephalopathy cases in Spain from 1998 to 2018 included in this study.

	Sporadic	Genetic			Acquired		Total
TSE^a^	sCJD^h^ *n* (%*)	gCJD^i^ *n* (%*)	FFI^j^ *n* (%*)	GSS^k^ *n* (%*)	atCJD^l^ *n* (%*)	vCJD^m^ *n* (%*)	Total cases
Cases	1,362 (89.0)	83 (5.4)	69 (4.5)	5 (0.3)	6 (0.4)	5 (0.3)	1,530
Median survival (months)	4.8	5.45	10.97	–	–	–	5.05
Age at onset, median (IQR^b^):	69 (62–75)	58 (51–64)	49 (45–57)	54 (38–Z–62)	48 (43–67)	47 (40–48)	68 (60–74)
Sex							
Male	619 (45.5)	39 (47)	38 (55.1)	1 (20.0)	3 (50.0)	1 (20.0)	701 (45.8)
Female	743 (54.6)	44 (53.0)	31 (44.9)	4 (80.0)	3 (50.0)	4 (80.0)	829 (54.2)
14-3-3 protein							
Positive	979 (71.9)	43 (73.5)	8 (11.6)	1 (20.0)	5 (83.3)	1 (20.0)	1,037 (67.8)
Negative	192 (14.1)	18 (21.7)	29 (42.0)	2 (40.0)	–	4 (80.0)	245 (16.0)
Polymorphism at codon 129							
MM^c^	469 (34.4)	43 (51.8)	47 (68.1)	–	5 (83.3)	5 (100)	569 (37.2)
MV^d^	149 (10.9)	30 (36.1)	16 (23.2)	–	–	–	195 (12.8)
VV^e^	153 (11.2)	3 (3.6)	–	3 (60.0)	–	–	159 (10.4)
EEG^f^							
Positive	754 (55.4)	44 (53.0)	3 (4.4)	1 (20.0)	1 (16.7)	1 (20.0)	804 (52.6)
Negative	546 (40.1)	32 (38.6)	59 (85.5)	2 (40.0)	5 (83.3)	4 (80.0)	648 (42.4)
MRI^g^							
Positive	501 (36.8)	42 (50.6)	–	–	–	1 (20.0)	544 (35.6)
Negative	535 (39.3)	25 (30.1)	43 (62.3)	2 (40.0)	5 (83.3)	2 (40.0)	612 (40.0)
Clinical onset							
Rapidly progressive dementia	752 (55.2)	35 (42.2)	17 (24.6)	–	2 (33.3)	2 (40.0)	808 (52.8)
Heidenhain	68 (5.0)	4 (4.8)	–	1 (20.0)	–	–	73 (4.8)
Psychiatric	50 (3.7)	7 (8.4)	15 (21.7)	–	–	2 (40.0)	74 (4.8)
Progressive dementia	98 (7.2)	8 (9.6)	4 (5.8)	–	1 (16.7)	1 (20.0)	112 (7.3)
Cerebellar	240 (17.6)	23 (27.7)	16 (23.2)	2 (40.0)	2 (33.3)	–	283 (18.5)
Extrapyramidal	44 (3.2)	4 (4.8)	1 (1.5)	1 (20.0)	–	–	50 (3.3)
Vascular	30 (2.2)	2 (2.4)	–	–	1 (16.7)	–	31 (2.0)
Period (year of diagnosis)							
1998–2003	339 (24.9)	19 (22.8)	24 (34.8)	0	3 (50.0)	0	385 (25.2)
2004–2008	340 (25.0)	20 (24.1)	15 (21.7)	1 (20.0)	1 (16.6)	5 (100.0)	382 (25.0)
2009–2013	351 (25.8)	26 (31.3)	18 (26.1)	3 (60.0)	1 (16.7)	0	399 (26.1)
2014–2018	332 (24.4)	18 (21.7)	12 (17.4)	1 (20.0)	1 (16.7)	0	364 (23.8)

^a^Transmissible spongiform encefalopaties, ^b^interquartile range, ^c^methionine/methionine, ^d^methionine/valine, ^e^valine/valine, ^f^electroencephalogram, ^g^magnetic resonance imaging, ^h^sporadic Creutzfeldt–Jakob disease, ^i^genetic Creutzfeldt–Jakob disease, ^j^Fatal Familial Insomnia, ^k^Gerstmann–Sträussler–Scheinker disease, ^l^accidentally transmitted Creutzfeldt–Jakob disease, ^m^variant Creutzfeldt–Jakob disease. *For the sake of simplicity, all percentages have been calculated vs. the value of total TSE cases (1,530), rather than vs. the total cases of each file at far right column.

In more than 90% of cases, valuable data for study purposes were available for type of TSE, sex, EEG, and year of diagnosis. Polymorphism at codon 129 was the least complete variable (60%). A total of 89% of cases were sporadic, and more than 50% of all cases had rapidly progressive dementia as clinical presentation.

The univariate and multivariate analyses of all cases showed that male sex, older age at onset, presence of 14-3-3 protein, typical MRI, and MM or VV in codon 129 polymorphism were associated with a shorter survival time. Sporadic CJD, typical EEG, and clinical presentation as rapidly progressive dementia displayed shorter survival times only in unadjusted comparisons but not after adjustment for sex, age at onset, codon 129 polymorphism, 14-3-3 protein, and typical MRI (Table 2). The mutations most frequently present in genetic cases from Table 2 were D178N, *n* = 73 (48.3% of total number of genetic cases with genetic analysis) and E200K, *n* = 68 (45%). Similar findings were obtained when only sporadic cases were analyzed (Table 3). However, typical MRI was not associated with survival in the unadjusted analysis.

**TABLE 2:** Univariate and multivariate Cox regression analysis for total cases (*n* = 1,530).

	Unadjusted HR^f^ (95% CI^g^)	Adjusted HR^f^ (95% CI^g^)
Male sex	1.26 (1.12–1.42)	1.24 (1.03–1.51)
Age at onset (years)		
0–49	Ref^h^	Ref^h^
50–59	1.77 (1.34–2.34)	1.53 (1.02–2.31)
60–69	1.83 (1.42–2.36)	1.46 (0.99–2.16)
70–79	2.37 (1.84–3.06)	2.04 (1.38–3.03)
≥80	2.33 (1.72–3.16)	2.46 (1.53–3.94)
Polymorphism at codon 129		
MM^a^	Ref^h^	Ref^h^
MV^b^	0.35 (0.28–0.43)	0.33 (0.25–0.44)
VV^c^	0.87 (0.71–1.05)	0.77 (0.59–1.00)
Positive 14-3-3 protein	2.01 (1.68–2.42)	1.76 (1.36–2.33)
Typical EEG^d^	1.55 (1.37–1.75)	1.04 (0.83–1.29)
Positive MRI^e^	1.19 (1.04–1.36)	1.31 (1.08–1.59)
Clinical onset		
Other symptoms	Ref^h^	Ref^h^
Rapidly progressive dementia	1.28 (1.14–1.45)	1.02 (0.85–1.24)
Established diagnosis		
Sporadic	Ref^h^	Ref^h^
Genetic	0.59 (0.48–0.72)	1.07 (0.76–1.50)

*Multivariate model includes all variables. ^a^Methionine/methionine, ^b^methionine/valine, ^c^valine/valine, ^d^electroencephalogram, ^e^magnetic resonance imaging, ^f^hazard ratio, ^g^confidence interval, ^h^reference.*

**TABLE 3:** Univariate and multivariate Cox regression analysis for sporadic cases (*n* = 1,362).

	Unadjusted HR^f^ (95% CI^g^)	Adjusted HR^f^ (95% CI^g^)
Male sex	1.30 (1.15–1.48)	1.39 (1.16–1.66)
Age at onset (years)		
0–49	Ref^h^	Ref^h^
50–59	1.67 (1.16–2.41)	1.91 (1.19–3.06)
60–69	1.66 (1.18–2.33)	1.74 (1.12–2.71)
70–79	2.08 (1.49–2.92)	2.24 (1.44–3.59)
≥ 80	2.04 (1.40–2.97)	2.59 (1.57–4.27)
Polymorphism at codon 129		
MM^a^	Ref^h^	Ref^h^
MV^b^	0.34 (0.27–0.44)	0.35 (0.26–0.47)
VV^c^	0.81 (0.66–0.99)	0.79 (0.62–0.99)
Positive 14-3-3 protein	1.97 (1.60–2.41)	1.86 (1.41–2.47)
Typical EEG^d^	1.39 (1.22–1.58)	0.99 (0.81–1.22)
Positive MRI^e^	1.12 (0.98–1.29)	NA
Clinical onset		
Other symptoms	Ref^h^	Ref^h^
Rapidly progressive dementia	1.18 (1.04–1.35)	1.08 (0.90–1.29)

*Multivariate model includes all variables. ^a^Methionine/methionine, ^b^methionine/valine, ^c^valine/valine, ^d^electroencephalogram, ^e^magnetic resonance imaging, ^f^hazard ratio, ^g^confidence interval, ^h^reference.*

The proportional hazards assumption was assessed in the final model. Type of TSE and polymorphism in the *PRNP* at codon 129 (overall and in sCJD) did not fulfill the proportional hazards assumption because the HRs of these variables changed over time. As the time of follow-up doubled, risk of death in genetic cases decreased by almost twofold. For total cases, a similar risk reduction was seen in MV and VV polymorphisms when compared to MM. For sCJD, risk of death decreased by almost twofold in MV cases and by almost fourfold in the VV polymorphism.

## Discussion

For TSEs as a whole, female sex, earlier onset, MV and VV polymorphisms at codon 129, and absence of 14-3-3 protein were observed to be associated with longer survival. Median age at onset decreased from the sporadic forms to gTSE and, lastly, to atTSE. Survival rose from sTSE to gTSE, in which it doubled before adjustment. Median survival at 12 months for sCJD, gCJD, and FFI was similar to that observed by Pocchiari et al. (2004) for the same categories at 12 months.

Survival decreased with advancing age at clinical onset, as noted in some reports (Puopolo et al., 2003; Pocchiari et al., 2004). It has been suggested that this type of decrease may be determined by age-related comorbidities or response to infection or CJD vascular-related pathogenesis, particularly in CJD forms of vascular onset (Pocchiari et al., 2004). Lower CJD case identification among the elderly with short survival due to lack of access to neurological diagnosis would bias our results toward higher survival. In our study, and in line with other authors, males registered shorter survival times than did females, although the reason for this remains unknown (Pocchiari et al., 2004; Iwasaki et al., 2015).

Codon 129 polymorphism constitutes a mortality risk factor (Kobayashi et al., 2015) that would act during the latency period (Uttley et al., 2020). The shorter survival in MM vs. MV polymorphism groups has been previously reported (Pocchiari et al., 2004). Although clinical presentation as dementia has been previously described as a mortality risk factor (Chen and Dong, 2016), this could not be confirmed after adjusting for confounding factors.

As 14-3-3 protein in CSF is considered to be a marker of rapid neuronal destruction, with levels changing with disease progression, our finding of shorter survival in cases positive to 14-3-3 protein may have been determined by their higher sensitivity when tested during late clinical course (Pocchiari et al., 2004; Torres et al., 2012). Disease duration was shorter when patients were positive to MRI, as mentioned above (Meissner et al., 2004). In contrast, patients with triphasic periodic complexes on the EEG, a finding most frequent in mid-to-late disease course (Franko et al., 2016), did not show shorter survival. This could be due to the low EEG voltage during late disease course (Collins et al., 2006).

Differences in survival between TSE entities appeared to diminish following adjustment for all study variables, which may indicate that many of these variables determine disease course. Similar patterns described for TSE in the majority of European Union member countries might suggest homogeneity of survival patterns across populations (Puopolo et al., 2003; Pocchiari et al., 2004). An element potentially responsible for a variation in our overall findings might be the proportion of sTSE and gTSE entities in our study, namely, 90 and 10%, respectively, which differed from those seen in Slovakia and Israel, where genetic forms predominate (de Pedro-Cuesta et al., 2006) but mimic those of some large populations, such as the United Kingdom, France, and Japan (Nozaki et al., 2010; Uttley et al., 2020).

As regards vCJD, Spain ranks third in terms of the number of cases, five, after the United Kingdom and France (Brandel and Knight, 2018). Median age at onset was higher than that described in other EuroCJD national populations (Puopolo et al., 2003; Pocchiari et al., 2004; Ladogana et al., 2005; Llorens et al., 2020; Uttley et al., 2020). The older age at onset of vCJD when compared to the United Kingdom and France (Brandel and Knight, 2018) might reflect a variation in the underlying etiological process. It would appear that both in Spain and in the large EuroCJD dataset, vCJD had the longest survival, whereas sCJD survival was the shortest, with gTSE occupying an intermediate position (Pocchiari et al., 2004).

Survival heterogeneity in sCJD and gTSE warrants particular attention. Our median figures of 5.2 months overall and 4.9 months for sCJD are similar to those described in Europe (Pocchiari et al., 2004) and China (Yang et al., 2020), and lower than those described in Taiwan, 13.5 months (Sun et al., 2020), and in greater detail in Japan, where the mean reached 17.4 months for all TSEs and 15.7 months for sCJD (Nagoshi et al., 2011; Iwasaki et al., 2015). Factors underlying the long survival of Japanese CJD patients are not well known, although some aspects of clinical management of akinetic mutism, such as tube feeding, have been suggested (Iwasaki et al., 2015). The high prevalence of the V180I mutation in Japan, 41%, in practice absent, 1%, in Western populations (Nozaki et al., 2010), exhibits a similarly higher survival (Hayashi et al., 2020) and constitutes one of the very few gTSE forms associated with slow progression. This feature is consistent with findings in Western studies for two reasons: firstly, our results show that differences in survival between sCJD and gTSE disappear after adjustment for age, among other variables; and secondly, the long survival in sCJD in populations with slowly progressing gTSE might be a natural history trait rather than the result of potentially different healthcare interventions. This feature fits recent proposals for conformational neurodegenerative disorders (NDDs), which suggest a causal link between genetic and sporadic forms (de Pedro-Cuesta et al., 2016b) and shared molecular mechanisms. In the case of TSEs, such a trait would imply that gTSE *PrP* might act as a transmission agent, a phenomenon suggested by the spatial clustering of high regional sTSE and gTSE incidences close to the Basque Country in Spain (De Pedro-Cuesta et al., 2021). The higher sCJD survival in Taiwan, where only eight genetic cases have been described (Sun et al., 2020) and particular genetic traits have been seen (Wang et al., 2007), merits further study. The fact that survival of gTSE in Spain is equal to that of sCJD, when age at onset, 129 codon polymorphism, and other variables are introduced into models (Brown and Mastrianni, 2010), would support similar survival in sTSE and gTSE forms. In sum, the two-facetted pattern of survival in sCJD, i.e., that in Japan and that shown by our and others’ results, would support the notion that gTSE and sTSE might share biological features resulting from similar PrP^Sc^ deposits, which, in the case of gTSE, determine an earlier onset by a disease-triggering mutation (de Pedro-Cuesta et al., 2016b).

The relationship between age at clinical onset and survival observed in the Spanish TSE cohort for sCJD and the difference observed between gTSE and sCJD raise the question of the presence of similar patterns for other NDDs classified by histochemical categories. While a full examination of the issue exceeds the scope of this paper, the view emerging from sCJD with lower survival and later onset appears to be the opposite to those seen for amyotrophic lateral sclerosis compared to fronto-temporal dementia reported by Steenland et al. (2010), and for α-synucleinopathies with rapid course and earlier onset, e.g., for multiple system atrophy compared to Parkinson’s disease (de Pedro-Cuesta et al., 2016b). Finally, the lower age at onset and higher survival of gTSE vs. sCJD forms seem to be a frequent feature seen in NDDs where family forms have been frequently denoted in keeping with the nature of their early-onset, e.g., early-onset parkinsonism in PD (Khodadadian et al., 2018).

Because TSEs constitute conformational NDDs, an interpretation of findings for sCJD and gTSE in our study and, in a broad perspective, from the so-called driver-model perspective, could be attempted. Drivers constitute groups of epidemiological features shared by protein disorders (de Pedro-Cuesta et al., 2016a), which provide an etiological framework for inference (de Pedro-Cuesta et al., 2016b). The simultaneous role as both a marker of disease course and a risk factor for NDDs, i.e., the APOe4 polymorphism for Alzheimer’s disease, proposed on the basis of well-known associations with genetic and vascular disorders, constitutes a fundamental feature of the driver model (de Pedro-Cuesta et al., 2016a). Consequently, certain variables associated with survival, such as those reflecting deposition secondary to geographically different *PRNP* gene mutations, might constitute risk factors for different forms of sporadic CJD in Western and Asian/Japanese populations.

This study may present limitations due to delays in notification or access to specific variables, such as glycotypes or the large accrual variation among different TSE groups. The failure to fulfill the proportional HRs assumption might be attributed to the very rapid disease course, with the majority of patients dying early after clinical onset. Such poorly explored phenomena would be present in most studies. Moreover, it cannot be ruled out that poor access to support measures for individual patients, documented in previous studies (Pocchiari et al., 2004; Staffaroni et al., 2019), may have affected their survival. This study’s strengths lie in its large population coverage, systematic data-collection, and compulsory reporting.

## Conclusion

In conclusion, TSE survival in Spain replicates the magnitude of that shown in Western countries, displays TSE entity-specific population patterns, and suggests the possible existence of underlying etiological mechanisms shared with other conformational NDDs. The fact that the same genetic or vascular factors might constitute risk factors for specific NDDs and predictors of clinical disease progression (de Pedro-Cuesta et al., 2016a,b) opens research avenues for inference between general and clinical epidemiology of NDDs, such as those suggested here for TSEs.

## Data Availability Statement

Unidentified data are available upon reasonable request. Requests to access these datasets should be directed to JA-I.

## Ethics Statement

Ethical review and approval was not required for the study on human participants in accordance with the local legislation and institutional requirements. Written informed consent from the participants’ legal guardian/next of kin was not required to participate in this study in accordance with the national legislation and the institutional requirements.

## Author Contributions

LT-R, TL-C, JA-I, FG, and JdP-C: concept and design and acquisition, and analysis, and interpretation of data. LT-R, JdP-C, FG, JA-I, and MC: drafting of the manuscript. LT-R, TL-C, JA-I, MC, FG, and JdP-C: critical revision of the manuscript for important intellectual content. TL-C and LT-R: statistical analysis. JdP-C and MC: obtained funding. JA-I and FG: administrative and technical, and material support. JdP-C: supervision. All authors contributed to the article and approved the submitted version.

## Conflict of Interest

The authors declare that the research was conducted in the absence of any commercial or financial relationships that could be construed as a potential conflict of interest.

## Publisher’s Note

All claims expressed in this article are solely those of the authors and do not necessarily represent those of their affiliated organizations, or those of the publisher, the editors and the reviewers. Any product that may be evaluated in this article, or claim that may be made by its manufacturer, is not guaranteed or endorsed by the publisher.
